# The impact of flooding on aquatic ecosystem services

**DOI:** 10.1007/s10533-018-0449-7

**Published:** 2018-05-11

**Authors:** Ceara J. Talbot, Elena M. Bennett, Kelsie Cassell, Daniel M. Hanes, Elizabeth C. Minor, Hans Paerl, Peter A. Raymond, Rodrigo Vargas, Philippe G. Vidon, Wilfred Wollheim, Marguerite A. Xenopoulos

**Affiliations:** 1 Environmental and Life Sciences Graduate Program, Trent University, Peterborough, ON Canada; 2 Department of Natural Resource Sciences and McGill School of Environment, McGill University, Ste-Anne-de-Bellevue, QC Canada; 3 Department of Epidemiology of Microbial Diseases, Yale School of Public Health, New Haven, CT USA; 4 Department of Earth and Atmospheric Sciences, Saint Louis University, St. Louis, MO USA; 5 Large Lakes Observatory and Department of Chemistry and Biochemistry, University of Minnesota Duluth, Duluth, MN USA; 6 Institute of Marine Sciences, University of North Carolina at Chapel Hill, Morehead City, NC USA; 7 School of Forestry and Environmental Studies, Yale University, New Haven, CT USA; 8 Department of Plant and Soil Science, University of Delaware, Newark, DE USA; 9 Department of Forest and Natural Resources Management, The State University of New York College of Environmental Science and Forestry (SUNY-ESF), Syracuse, NY USA; 10 Department of Natural Resources and the Environment, University of New Hampshire, Durham, NH USA; 11 Department of Biology, Trent University, Peterborough, ON Canada

**Keywords:** Ecosystem services, Extreme floods, Freshwater, Rivers, Floodwaters, High discharge, Floodplains, Natural floods, Ecological functions

## Abstract

Flooding is a major disturbance that impacts aquatic ecosystems and the ecosystem services that they provide. Predicted increases in global flood risk due to land use change and water cycle intensification will likely only increase the frequency and severity of these impacts. Extreme flooding events can cause loss of life and significant destruction to property and infrastructure, effects that are easily recognized and frequently reported in the media. However, flooding also has many other effects on people through freshwater aquatic ecosystem services, which often go unrecognized because they are less evident and can be difficult to evaluate. Here, we identify the effects that small magnitude frequently occurring floods (< 10-year recurrence interval) and extreme floods (> 100-year recurrence interval) have on ten aquatic ecosystem services through a systematic literature review. We focused on ecosystem services considered by the Millennium Ecosystem Assessment including: (1) supporting services (primary production, soil formation), (2) regulating services (water regulation, water quality, disease regulation, climate regulation), (3) provisioning services (drinking water, food supply), and (4) cultural services (aesthetic value, recreation and tourism). The literature search resulted in 117 studies and each of the ten ecosystem services was represented by an average of 12 ± 4 studies. Extreme floods resulted in losses in almost every ecosystem service considered in this study. However, small floods had neutral or positive effects on half of the ecosystem services we considered. For example, small floods led to increases in primary production, water regulation, and recreation and tourism. Decision-making that preserves small floods while reducing the impacts of extreme floods can increase ecosystem service provision and minimize losses.

**Electronic supplementary material:**

The online version of this article (10.1007/s10533-018-0449-7) contains supplementary material, which is available to authorized users.

## Introduction

**Fig. 1:**
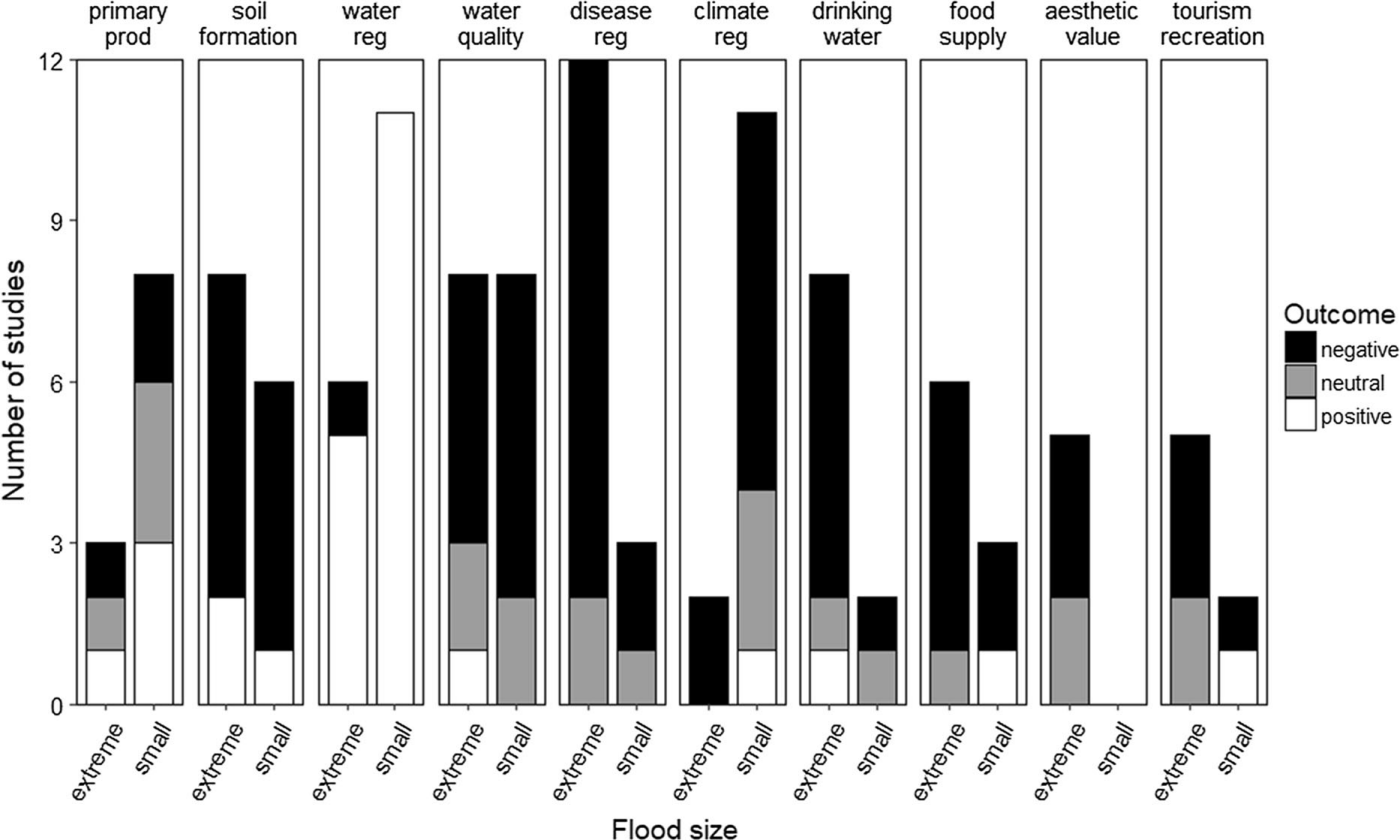
Number of studies resulting from a systematic literature review with negative, neutral, and positive outcomes on ten aquatic ecosystem services following small and extreme floods

Flooding is usually considered a significant natural hazard causing disease, damage and loss to life, property, and infrastructure as well as disruption of public services. For example, floods can cause dangerous landslides (Hong et al. 2007), loss of crops and livestock (Atta-ur-Rahman 2011), disruption of normal drainage systems (Ogden et al. 2011), spillage of raw sewage and animal waste, and accelerated discharge of industrial and urban toxic materials (Euripidou and Murray 2004) and nutrients into waterways (Hubbard et al. 2011). Because of their dramatic effects on people and infrastructure, the effects of flooding on aquatic ecosystems are often viewed as negative; however, this is not always the case. Flooding can also provide many benefits, including recharging groundwater, increasing fish production, creating wildlife habitat, recharging wetlands, constructing floodplains, and rejuvenating soil fertility (Poff 2002). Since the effects of flooding on aquatic ecosystems can be both negative and positive, ecosystem services should also exhibit a mix of negative and positive outcomes resulting from flooding (Terrado et al. 2013). However, it is still unclear how floods of different magnitudes could affect gains or losses in ecosystem services (“the benefits people obtain from ecosystems” MA 2005) or how individual ecosystem services will be affected (Fig. 1).

**Fig. 2:**
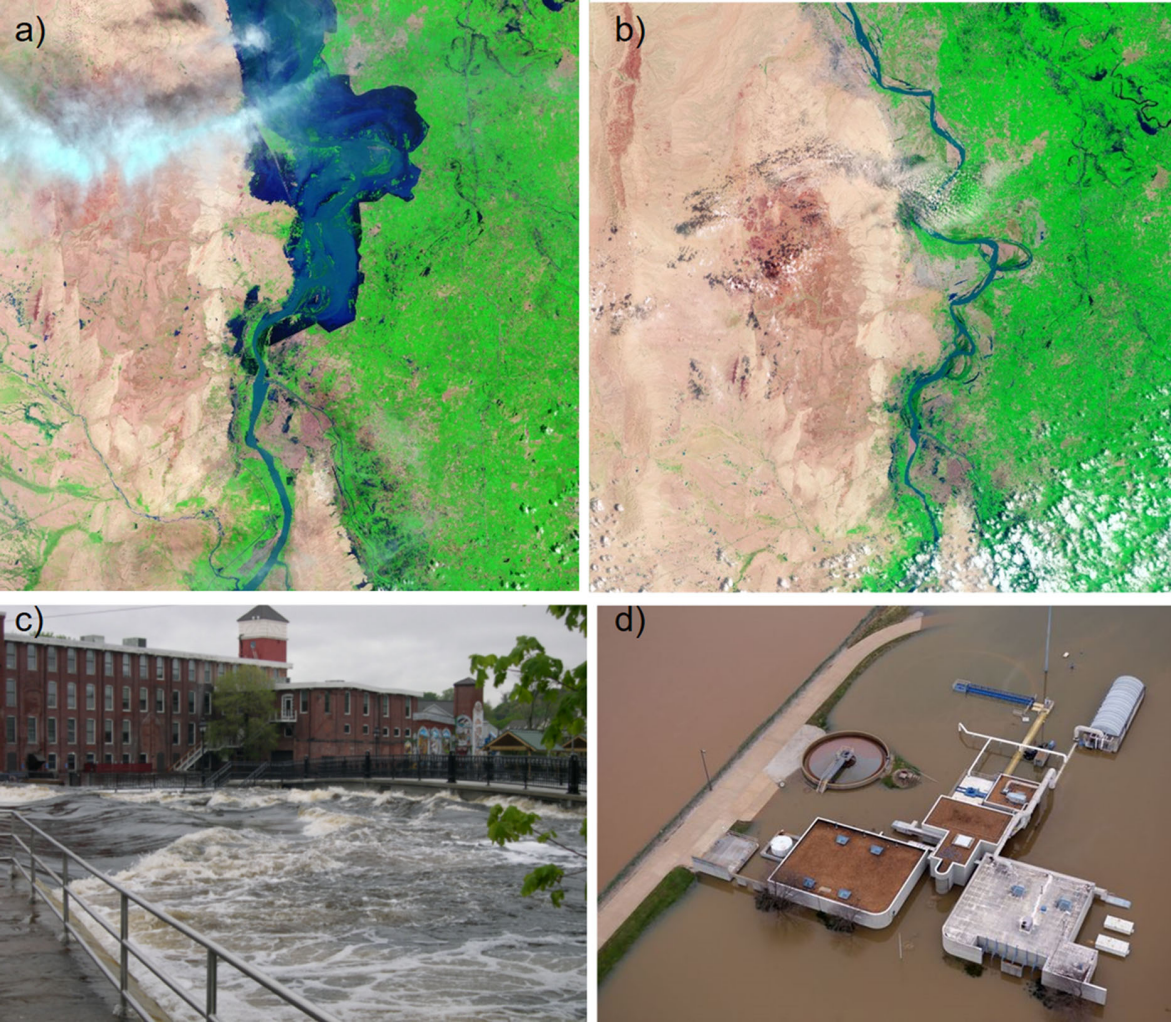
Photos of flooding taken from different perspectives. Satellite photos of extreme flooding (**a**) and seasonal flooding (**b**) on the Indus River, Pakistan, ground level photo of extreme flooding on the Ipswich River, Massachusetts, USA (**c**) and aerial photo of extreme flooding engulfing a sewage treatment plant on the Meramec River, Missouri, USA (**d**). Image sources: NASA Earth Observatory, https://earthobservatory.nasa.gov/IOTD/view.php?id=45393 (**a**, **b**), Wilfred Wollheim (**c)**, David Carson, St Louis Post-Dispatch (**d**)

Floods occur when low-lying areas that are typically dry become temporarily inundated with water outside of their normal confines (Rojas et al. 2013). Flooding accounts for one-third of natural disasters and affects more people than any other type of disaster (Sivakumar 2011). Flood-related impacts are expected to worsen due to global environmental change with flood risk increasing by 187% from increasing temperature in the HadCM3 climate model (Arnell and Gosling 2016). Flood magnitude is also expected to increase due to intensified water cycling resulting from as little as a 1.5 °C global average temperature increase (Alfieri et al. 2017). However, all floods are not created equal and the causes and consequences of individual floods are often unique. Floods can be seasonal as in the case of spring snowmelt or monsoon rains or they can occur randomly via several other mechanisms such as ice jams, storm surges, and heavy precipitation (Fig. 2a–c). Heavy precipitation accounts for about 65% of river floods (Douben 2006), but northern latitude areas with snow cover are also vulnerable to flooding caused by snowmelt and sometimes exacerbated by rain events (Kundzewicz et al. 2014). Flood events have been further characterized based on magnitude, frequency, duration, and volume (Burn and Whitfield 2016). These characteristics are important for determining the effects of floods on both aquatic ecosystems and the people who benefit from them. For example, flood magnitude can determine the amount of groundwater recharge or the extent of home and infrastructure damage during flooding. Flood magnitude is only one aspect of predicting flood impacts on aquatic ecosystems and ecosystem services. Ecosystem conditions prior to flooding are potentially equally as important as flood characteristics for determining ecosystem response to a flood event.

Rivers need floods to create unique habitat and support biological productivity and biodiversity. The Flood Pulse Concept states that predictable seasonal floods are beneficial for riverine systems and can influence biotic composition, nutrient transport, and sediment distribution but unpredictable floods may be disruptive for aquatic organisms (Junk et al. 1989). Additionally, many aquatic ecosystems have reduced resilience to future extreme events such as flooding due to human activities that include urban development and farming on floodplains, river flow disruptions, and pollution (Woodward et al. 2016). These activities increase the likelihood that floods become catastrophic events especially from the perspective of “benefits” obtained from ecosystems. The specific effects of flooding on aquatic ecosystems and their services are not well understood, but the importance of flooding for maintaining ecological functions in rivers has been recognized (Peters et al. 2016). Most of the research on flooding takes advantage of fortuitous events and thus often lacks pre-flood reference data (Poff and Zimmerman 2010). This relatively sparse evidence on how flooding and changes in hydrology impact aquatic ecosystems drives a large amount of environmental flow management (Acreman et al. 2014) and flood-related research.

Using an ecosystem service approach can help advance our understanding of the impacts of flooding on aquatic ecosystems and how future changes in flood magnitude will change the availability of aquatic ecosystem services. People have taken advantage of various ecosystem services for over 10,000 years (Fisher et al. 2008), making them integral to society. In fact, the estimated global value of all ecosystem services in 2011 was $125 trillion/year (Costanza et al. 2014). There are many studies that evaluate the effects of disturbances on ecosystem services, but most of these studies focus on terrestrial systems and there are few that look at aquatic ecosystem services (Grizzetti et al. 2016). Furthermore, there are even fewer studies that integrate the effects of hydrologic changes (Terredo et al. 2013). Aquatic ecosystems provide many services such as drinking water, soil formation, primary production, and areas for recreation or tourism, but flooding can impact the availability of these services. We expected to find that flood magnitude plays a role in determining whether aquatic ecosystem services are lost or gained following flood events. We expected that small floods would lead to gains in aquatic ecosystem services, while extreme floods would lead to losses. If ecosystem services respond to small and extreme magnitude floods differently, then current flood mitigation strategies may be detrimental to aquatic ecosystem services. Common flood mitigation activities such as damming and flood barrier implementation restrict the occurrence of small floods but are often unable to mitigate extreme floods (Alfieri et al. 2016).

In this study, we examined the societal pros and cons of various flooding events by evaluating their effects on aquatic ecosystem services. We used our current understanding of ecosystem services and flood impacts on aquatic ecosystems to identify gains and losses in ecosystem services resulting from flood events of different magnitudes. We completed a systematic literature review on a subset of 10 aquatic ecosystem services thought to be directly influenced by flooding to determine whether small versus extreme floods cause gains or losses in these services (Table 1). The ecosystem services included represent a variety of service types (i.e., provisioning, supporting, cultural, and regulating) from the Millennium Ecosystem Assessment framework (MA 2005) to create a holistic view of the ecosystem response to flooding. We also compared the influences of small versus extreme magnitude floods on each of the 10 ecosystem services to distinguish between normal (often seasonal) flooding and rare extreme events that may impact aquatic ecosystems differently. We hypothesized that small floods would enhance ecosystem service provisioning compared to large floods, which we expected would have more negative effects on ecosystem services. Ultimately, our study can be used to inform effective flood protection strategies that can mitigate the undesirable consequences of flooding while preserving aquatic ecosystem services. Decision makers may use the demonstrated importance of small versus extreme floods for ecosystem services to better manage for variable flows, including small and occasional extreme floods. Because ecosystem services are derived from well-functioning ecosystems, managing for ecosystem services may simultaneously benefit people and aquatic ecosystems.

**Table 1:** Ecosystem services with indicators used to capture ecosystem service changes, indicator units, process linking ecosystem service with flooding, and ecosystem service type as defined in the Millennium Ecosystem Assessment

Ecosystem service	Indicator	Unit	Process	Type
Primary production	NPP, GPP	mg C/m^3^/time	Changes in nutrients and physical conditions impact NPP/GPP	Supporting
Soil formation	Erosion, accumulation volume	m^3^	Sediment deposition on shores/more sediment transport in water	Supporting
Water regulation	Groundwater and aquifer volume or height	m^3^, m	Water retained in ecosystem for some anthropogenic use (drinking, irrigation, etc.)	Regulating
Water quality	Water nitrogen and phosphorus concentration	µg/L, mg/L	Increased nutrient transport	Regulating
Regulation of human disease	Odds ratio	None	Release of disease-causing agents from sediment or overflowing sewer systems	Regulating
Climate regulation	Methane and carbon dioxide release	g CH_4_/time	Changes in aerobic/anaerobic microbial processes that influence organic matter decomposition	Regulating
Drinking water	Total coliform, metal concentrations	cfu/mL, mg/L	Bacteria and metals mobilized by floodwaters and enter drinking water sources	Provisioning
Food supply	Crops damaged, change in fish catch	None	Crops destroyed by physical impacts of floodwater, changes in fish distribution and abundance	Provisioning
Aesthetic value	Housing value discount	$	Damage and risk of flooding reduce desire to live near water	Cultural
Recreation and tourism	Willingness to visit recreation area, revenue lost	$	Algal bloom, unsafe water levels, debris in water, lack of infrastructure to travel to destination	Cultural

## Methodology

The Millennium Ecosystem Assessment (MA) aimed to address how ecosystem change can affect ecosystem services and their beneficiaries and to find a scientific way to ensure sustainable use and conservation of these services (MA 2005). Many ecosystem service frameworks have been developed since the MA such as Final Ecosystem Goods and Services Classification System (FEGS-CS; Landers and Nahlik, 2013), Stressor–Ecological Production function–final ecosystem Services (STEPS; Bell et al. 2017), and Ecosystem Service Profile (ESP; Paetzold et al. 2010). These frameworks and others typically focus on final services (services that people use directly) and emphasize economic valuation, which was not the goal of our analysis. Additionally, none of these frameworks are widely used (Nahlik et al. 2012). Therefore, we chose to use the MA framework to structure our analysis because it is commonly used to evaluate ecosystem services and is flexible enough to capture many types of services. We used a group of 10 ecosystem services identified by the MA framework spanning the following four MA categories; (1) regulating services (benefits resulting from the regulation of ecosystem processes), (2) provisioning services (services that provide a product), (3) supporting services (services that aid in the production of all other ecosystem services), and (4) cultural services (nonmaterial benefits) (MA 2005) (Table 1). Supporting services are ecosystem functions and processes, which aid in the production of other services (Brauman et al. 2007). For example, soil formation provides one of the materials necessary for agriculture, contributing to the provisioning service of food supply. Since the MA was completed, the ecosystem services concept has evolved and supporting services are now typically considered ecosystem functions rather than benefits or ecosystem services (Haines-Young and Potschin 2010). However, we included supporting services in our analysis in order to capture a larger range of possible aquatic ecosystem responses to flooding. In contrast, provisioning services provide a material product that can be harvested or collected and then traded in markets (Brauman et al. 2007). Regulating services regulate ecosystem processes, providing a suitable environment for people to live in (Braat and de Groot 2012). Cultural services are also non-material goods. They provide sensory experiences that enhance quality of life such as areas for recreation and tourism and aesthetic value. Ecosystem services can be assessed either by quantifying biophysical changes or by assigning a dollar value to those changes (Braat and de Groot 2012). We used indicators of ecosystem service changes derived from variables measured in studies collected during our literature review to determine gains and losses in ecosystem services after flooding. We found that a variety of indicators or variables were used to report changes in the same ecosystem service; therefore, we included as many commonly reported indicators as possible. Because each flooding event is context dependent (e.g., antecedent conditions, soil conditions, ambient water conditions, etc.) and pre-flood data was often lacking from studies we could not quantify a general response to floods. Instead, we provide a *general pattern* (rather than a quantitative change) of ecosystem service changes in response to flooding.

We performed a systematic literature review to locate existing research on the effects of flooding on ecosystem services. We obtained published articles from Web of Science from 1980 to 2017 and summarized them. We focused upon the impacts of river basin flooding rather than flooding involving seawater intrusion or saltwater flooding, but studies included contained a variety of flood-generating mechanisms such as monsoons, cyclones, snowmelt, storm surges, and heavy precipitation. We chose to use flood return interval to characterize floods as either small or extreme because it is commonly present in the published literature. Other flood characteristics such as duration and frequency are also important for determining the effects of flooding but were rarely reported in published literature and therefore not explicitly considered in this study. We aimed to include both small floods (defined as < 10-year recurrence interval) and extreme floods (> 100-year return interval). This was a challenge because the impacts of small and seasonal floods are often not reported (Douben 2006). Therefore, the analyses of extreme flood impacts on ecosystem services are more complete. We searched for each ecosystem service individually. Each search began with the terms “flood” OR “flooding” OR “floods”. Then, specific terms related to each indicator were added. For example, the terms “(“flood” OR “flooding” OR “floods”) AND river AND (“outbreak risk” OR disease)” were used to search for literature relevant to human disease regulation. We followed-up the initial literature search with searches aimed at finding additional studies on small floods. We used the same ecosystem service-specific terms but replaced “flood” with “high discharge” and “storm”. This increased the number of results returned during searches, but many studies were excluded because they did not report overbank flow or inundation, thus not allowing us to accurately characterize the flood. All studies with abstracts containing information about a specific flood or storm event and a variable representing an ecosystem service were downloaded. We screened each of these studies one additional time to identify studies, which included a quantitative measure of the flood impact such as before and after measures of the same variable (e.g. Table 2). These initial literature results were augmented by further targeted searches on specific services and other work cited in the initially identified papers.

**Table 2:** Examples of quantitative changes in climate regulation and disease regulation ecosystem service indicators, where pre-flood, post-small flood, and post-extreme flood values were derived from the same study

Ecosystem service	Location	Indicator	Pre-flood value	Post-small flood	Post-extreme flood	Reference
Climate regulation	Danube River, Austria	CH_4_ flux (µmol/m^2^/h)	72.2	77.4	303.2	Sieczko et al. (2016)
Regulation of human disease	China	Odds ratio	1.00	1.14	1.28	Gao et al. (2016)

**Fig. 3:**
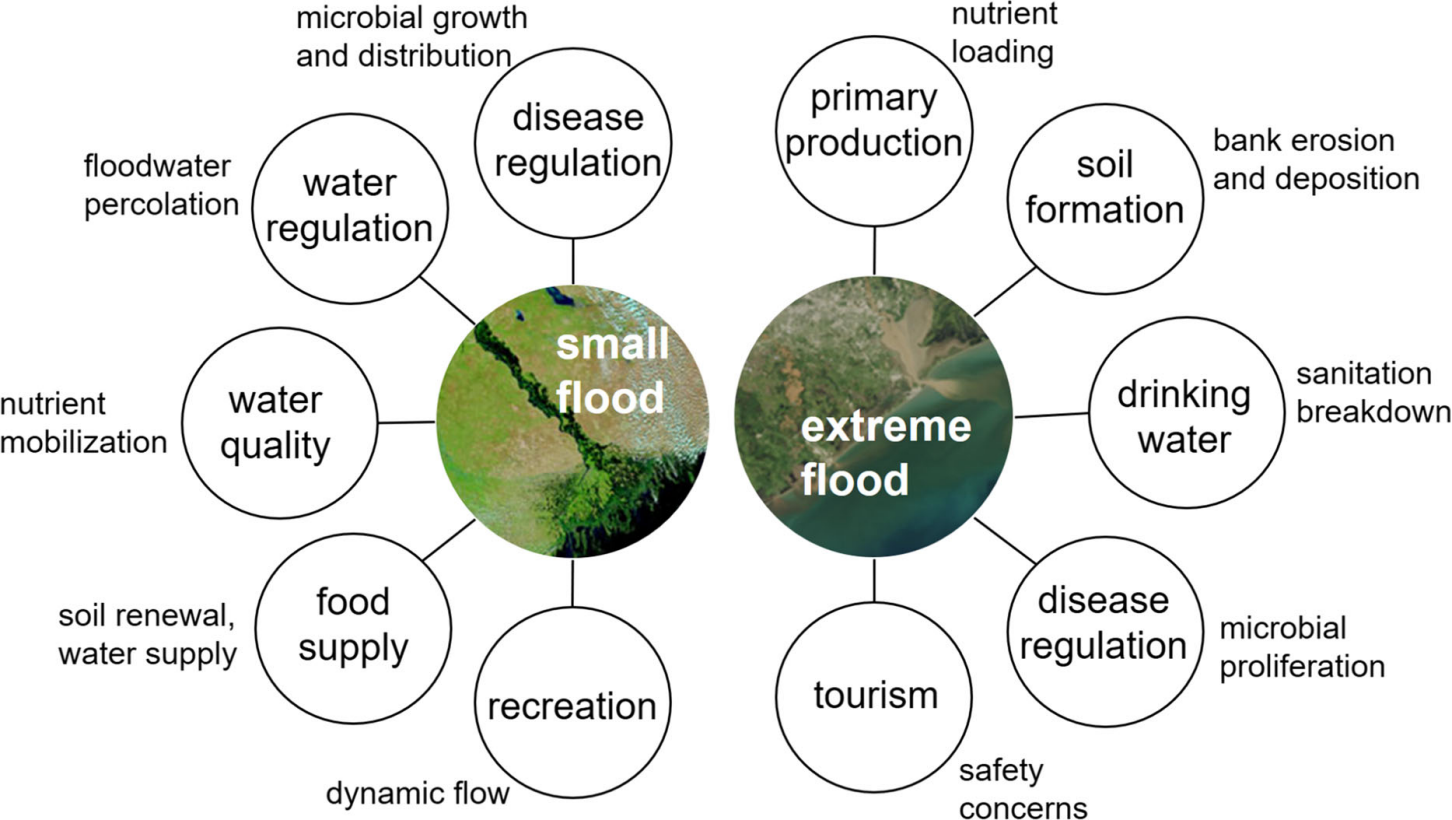
Processes linking small and extreme floods to changes in aquatic ecosystem services. Image sources: NASA Earth Observatory, https://earthobservatory.nasa.gov/NaturalHazards/view.php?id=14932&eocn=image&eoci=related_image (left) and https://earthobservatory.nasa.gov/IOTD/view.php?id=90703 (right)

This resulted in 117 studies after the literature search given described constraints. Each ecosystem service was represented by an average of 12 ± 4 studies. In general, the literature reported negative effects associated with flooding. Flooding is commonly perceived as detrimental and most studies tend to focus on the negative impacts of floods rather than the positive impacts. This bias may have skewed our results toward greater ecosystem service losses, but we were still able to identify ecosystem services which benefit from flooding. Ecosystem service availability varied with flood magnitude (Fig. 1; Table 3). Both small and extreme floods generally decreased the availability of most ecosystem services. However, extreme floods caused a greater number of ecosystem service losses than small floods (Table 3). Extreme floods were beneficial for groundwater and aquifer recharge and therefore were positive for these services. Small floods were important for improving access to food and recreation as well as beneficial for water regulation and primary production. The impacts of floods on ecosystem services were also related to initial physical, chemical, and biological conditions within the ecosystem and its location. These complex interactions made it difficult to attribute changes in ecosystem services to specific flood events. For example, post-flood changes in primary production varied because of temperature, light, and nutrient conditions. Additionally, there was some variation within individual ecosystem services which made assigning a negative, neutral, or positive outcome difficult. However, we were able to identify many of the possible underlying mechanisms that were responsible for ecosystem service outcomes post-flood from reviewed literature (Fig. 3). Below we describe each ecosystem service and its connection to flooding in more detail.

**Table 3:** Summary of the impacts of small and extreme floods on ecosystem service gains and losses

Ecosystem service	Gains or losses (+/−/0)	
	Small flood	Extreme flood
Primary production	+	+
Soil formation	−	−
Water regulation	+	+
Water quality	−	−
Regulation of human disease	−	−
Climate regulation	0	−
Drinking water	0	−
Food supply	−	−
Aesthetic value	NA	−
Recreation and tourism	+	−

Gains are expressed as “+”, losses as “−“, and neutral effects as “0”

## Supporting services

### Primary production

Hydrology is known to influence primary production by affecting water clarity, oxygen, pH, and nutrient concentrations (Lindholm et al. 2007). Floods may initially inhibit primary production while water is high but nutrients mobilized during storms may be held and processed in ecosystems later, when water levels return to normal (Paerl et al. 2011). Small seasonal floods contribute nutrients to aquatic ecosystems and can stimulate primary production (Junk et al. 1989), a process that is especially important in nutrient-poor oligotrophic systems. Increased primary production can then support aquatic food webs, providing a food source for consumers (Alford and Walker 2013). However, larger floods can transport excessive nutrients and potentially stimulate excessive primary production (i.e., eutrophication) or alter primary producer community composition, causing unfavorable species to dominate. Recently, increases in primary production have been attributed to increased phosphorus (P) and nitrogen (N) loading associated with flood events (Paerl et al. 2016). For example, flooding in the Lake Winnipeg catchment increased phytoplankton biomass and the phytoplankton community shifted to include more cyanobacteria (McCullough et al. 2012). Heavy rainfalls in the Lake Erie basin caused significant P loading and resulted in the largest algal bloom in the lake’s history (King et al. 2017). Harmful algal blooms (HABs) such as those which occurred in Lakes Winnipeg and Erie cause several problems for people who rely on these water bodies for drinking water and recreation. HABs include cyanobacteria which produce toxins that must be removed from drinking water supplies (Hitzfeld et al. 2000). HABs also lead to poor aesthetics, which adversely affect tourism and recreation activities, with detrimental impacts on local economies such as those around Lake Erie (Watson et al. 2016). Primary production benefits aquatic ecosystems up to a certain tipping point, when HABs can dominate and negate these benefits (Paerl et al. 2016). Therefore, increased primary production post-flood is considered an ecosystem service net gain but if primary production is excessive then flooding results in a net loss. Additionally, if a flood event decreases primary production, then it is considered a net loss.

Our literature review uncovered no consistent patterns of post-flood primary production responses. Both increases and decreases in primary production after flooding were reported. One study reported higher gross primary productivity (GPP) after a small flood (e.g. Lindholm et al. 2007), but other studies reported lower GPP post-flood (e.g. Uehlinger 2000; Uehlinger et al. 2003). Chlorophyll *a* (used as a surrogate for primary production) concentrations were also observed as decreasing after small floods (e.g. Rodrigues et al. 2002; Weilhoefer et al. 2008). Differential responses in primary production are likely the result of differences in nutrient supply, light penetration, and flushing rates of impacted ecosystems (Paerl et al. 2014a, b, 2016). Additionally, post-flood increases in nutrient supply must occur simultaneously with sufficient light penetration to cause increases in primary production. Minor et al. (2014) found that increases in post-flood P did not increase primary production because light was limited by increases in total suspended solids (TSS) and chromophoric dissolved organic matter (CDOM). The two studies reporting on the effects of extreme flooding on primary production also contained mixed results. Silva et al. (2013) reported that extreme flooding increased net primary productivity (NPP). The second study reported that chlorophyll *a* did not change after a “high magnitude” flood (Weilhoefer et al. 2008). In addition to providing nutrients, freshwater discharge resulting from flood events modulates the rate of flushing (or water residence time) of receiving waters. If flushing rates exceed algal growth rates, large flood events could reduce algal biomass, regardless of nutrient enrichment (Peierls et al. 2012; Paerl et al. 2014b). We therefore cannot consistently conclude whether flooding increases or decreases primary production and algal biomass since these indicators are highly dependent on other, interacting variables such as nutrient enrichment, water clarity, flushing rates, and grazing. However, the potential for large algal blooms occurs after flooding when nutrients are high and water residence time is long enough to allow blooms to form and accumulate (Paerl et al. 2016).

### Soil formation

Soil formation provides an essential service by regenerating river banks, wetlands, and flood-plain farmland. Flooding causes over bank flow and changes the rate of sediment deposition and erosional processes occurring between the river and floodplain (Junk et al. 1989). Flooding can cause river bank erosion and collapse, as well as upland erosion and incision, leading to landslides in areas with hillslopes and mountainous terrain (Larsen and Montgomery 2012) which pose threats to people (e.g. Kala 2014). Alternatively, flooding can improve soil formation by depositing sediment on floodplains, which recharges farmland soils and increases suitability for farming (Ogbodo 2011). Therefore, the net positive or negative impacts of flooding on soil formation depend on where erosion and deposition occur and the volume of sediment transported.

The influence of a flood event on erosion and accumulation is related to the flow peak magnitude (Julian and Torres 2006). Extreme floods increase erosion, but up to 70% of eroded sediment can be re-deposited within the catchment (Morche et al. 2007). Such re-deposition events are important in maintaining coastal forests and wetlands (e.g. Nyman et al. 1995; Bryant and Chabreck 1998; Shaffer et al. 2016) that act as key buffers against storm surges, biogeochemical filters for water entering coastal oceans and large lake systems, and critical nursery sites for important fisheries (e.g. Barbier et al. 2011). Therefore, soil erosion processes are spatially dynamic and the negative effects of erosion in certain locations, such as river banks or hill slopes, may enhance soil formation in other areas of a catchment, such as floodplains (Pearson et al. 2016). Such effects can be strongly exacerbated by land use practices, and over time, can lead to both improved farming locations and detrimental, even catastrophic flooding within the same river basin, as illustrated by the Yellow River catchment in China over the past 7000 years (Rosen et al. 2015). We found that extreme flooding caused substantial amounts of soil to be eroded in all studies. In one study, the volume of soil eroded during an extreme flood was 87% of the total eroded volume during a period of six years (Carroll et al. 2004). Another study reported over 1.4 million m^3^ of soil was eroded from a catchment in New Zealand (Fuller 2008).

Small floods also influence soil formation, although their effects are less dramatic than extreme events. Some studies, such as one by Dewan et al. (2017), have shown that discharge and erosion are correlated so small floods likely cause a small amount of erosion. In addition to less erosion, small floods lead to less sediment accretion on river banks. Stromberg et al. (1993) compared sediment accretion on banks following flood events with 2, 5, and 10-year flood recurrence intervals in Arizona, USA. They found that soil accretion generally increased with flood magnitude, but sediment accretion was similar in the 2 and 5-year floods compared to the 10-year flood (Stromberg et al. 1993). Studies reporting the effects of multiple small events were more common than those reporting on single flood events. An example of a multiple-event study is by Leyland et al. (2017), where they found that the mean rate of soil erosion was 4 times larger than the mean rate of soil accretion during the 2014 monsoon season in the Mekong River catchment. Multiple-event studies are difficult to compare because some include an entire flooding season, while others include a few flood events. Therefore, more studies on small individual flood events would be beneficial for assessing the impacts of small floods on soil formation.

## Regulating services

### Water regulation

Flooding is important for recharging underground water sources and recharge that results from flooding is especially beneficial during dry seasons when groundwater is the main source of freshwater in areas that experience pronounced wet and dry seasons (Kazama et al. 2007). In most cases, floodwaters are beneficial to recharge groundwater but this equation is changing with population growth. Demand for drinking water and water for irrigation will increase with population growth (Singh et al. 2014) and put further stress on surface water supplies that are already extensively exploited, causing people to rely more on groundwater (Wada et al. 2014; FAO 2016). As a result, human populations deplete underground water stores through extraction for irrigation and, to a lesser extent, drinking water. The need for irrigation to supply water to crops will also likely increase in areas where global environmental change is expected to increase temperatures and change precipitation patterns and where people are converting natural land covers to agricultural land (Taylor et al. 2013).

The effects of flooding on water regulation vary depending on floodplain conditions and natural hydrologic variability. For example, there is evidence that groundwater recharge is dependent on flood duration (Benito et al. 2010; Dahan et al. 2008) and floodplain land use (Keilholz et al. 2015). Additionally, inundation area determines how much floodwater infiltrates groundwater stores and larger inundation areas lead to more groundwater recharge. Therefore, flood mitigation strategies that reduce inundation area are detrimental to groundwater recharge processes (Kazama et al. 2007). However, groundwater levels that increase during flooding and extend above riverbeds or the soil surface can also contribute to more extreme flooding (e.g. Gotkowitz et al. 2014). Groundwater flooding can last longer than riverine overbank flooding and possibly inundate basements, agricultural land, and roads (Hughes et al. 2011). Therefore, it is optimal when groundwater is recharged but not to the point of overfilling during floods.

In our review of past flooding events, groundwater recharge increased with flooding in all 13 studies. Most studies reported that extreme floods contributed more water to underground stores than small floods, but one study showed that smaller floods contributed a disproportionately large amount of water to groundwater stores (Aksoy and Wittenberg 2015). Extreme floods contributed high volumes of water to groundwater stores. For example, an extreme flood increased the groundwater level by 0.8 m, causing additional above ground flooding (Gotkowitz et al. 2014). Additionally, Wang et al. (2015) reported that an extreme flood event increased groundwater depth by 3.24 m. Small floods occurring seasonally were also capable of supplying substantial amounts of water. For example, one seasonal flood increased groundwater level by more than 0.5 m (Amiaz et al. 2011). In another study, spring flooding contributed 40% of water to the annual groundwater recharge (Ray et al. 2002). Therefore, both extreme, rare floods, and small floods occurring seasonally lead to increased water volume in underground water stores and improved water regulation.

### Water quality

Flood events have contrasting effects on water quality. Increased terrestrial runoff from both surface and subsurface flow paths mobilize more dissolved nutrients on the landscape and reduce residence time in potential terrestrial sinks compared to water entering during base flow (Buda and Dewalle 2009; Bende-Michl et al. 2013). As a result, more nutrients are loaded into surface waters. However, while fluxes of dissolved constituents always increase during storms, concentrations show varied responses and may actually decline due in part to dilution during high flow events (Goodridge and Melack 2012; Carey et al. 2014; Wollheim et al. 2017). In contrast, sediment concentrations and dissolved organic matter concentrations generally increase during storms, so that fluxes will increase at greater rates than discharge (Raymond and Saiers 2010; Williams 1989). Total suspended solids (TSS) increases are further exacerbated in urban and agricultural catchments (Pizarro et al. 2014), while dissolved organic carbon (DOC) tends to increase more in forests and wetland systems (Huntington and Aiken 2013). TSS and DOC have direct drinking water quality implications, while the impact of nutrients is often more indirect through ecosystem function such as stimulating primary production and creating suitable habitat and resources for aquatic organisms. Thus, extreme flood events are likely to exacerbate water quality issues, particularly in watersheds dominated by anthropogenic land uses.

Water quality is further influenced by transport, mixing, and dilution within the river network (Hale et al. 2014). As a result, the spatial pattern of water quality degradation depends on the extent of the extreme event relative to pollution sources, the amount of runoff from clean water generating regions, and their spatial connectivity, which is also a question of scale. For example, a pollution source located downstream may be considerably diluted during extreme events due to massive upstream water inputs, as is evident in the Merrimack R. watershed, New Hampshire, USA (Samal et al. 2017). Total flux still increases, but concentrations can decrease due to dilution, so water quality impacts will depend on whether total flux or concentrations are more important for determining effects of pollutant changes.

Finally, aquatic transformations within the river network may affect water quality. Transformations include retention (e.g., settling of sediments, assimilation of nutrients) or permanent removal (e.g., denitrification). This regulating ecosystem service is strongly affected by flow (Doyle 2005; Hale et al. 2014; Wollheim et al. 2008; Wollheim et al. This Issue). Generally, as flow increases, the ability to regulate downstream dissolved fluxes declines. However, this decline is a function of watershed size (length of flowpaths within a river network), the distribution of sources within the watershed, the abundance of lakes, reservoirs and wetlands, as well as connectivity with floodplains (Mineau et al. 2015; Wollheim et al. This Issue). Extreme floods are likely to connect flowing waters with floodplains where soils high in organic matter may remove nutrients (Ensign et al. 2008). Models suggest that there is an optimal level of inundation for nutrient removal at network scales, most likely when flood waters are shallow and widely dispersed, and before waters become deeper (with less contact with sediments) (Noe and Hupp 2009). However, this has not been empirically demonstrated. Nevertheless, floodplains are likely to regulate downstream fluxes where they occur. Anthropogenically-driven modifications such as levee building disconnect channels from floodplains, and thereby remove this function. As a result, storms transport more material downstream, potentially degrading water quality.

### Regulation of human disease

Extreme flooding is a leading cause of weather related infectious disease outbreaks (Cann et al. 2013) and can overwhelm or damage sanitation systems, lowering the quality of water treatment, and in more extreme cases allowing sewage, industrial waste, and agricultural waste to mix with drinking water (Fig. 2d). Increases in disease after floods range from waterborne infections such as cholera and hepatitis A, to pathogens with more complex life cycles and transmission pathways like schistosomiasis and malaria. Flooding can disproportionately affect populations that are already at increased risk of disease due to poverty, poor sanitation and housing, and limited access to healthcare systems. Quantifying disease occurrence attributable to floods is complicated by the long lag periods between the flood and disease presentation, as well as differences by location and population. Despite these difficulties, multiple studies have revealed associations between flooding and increases in disease.

Pathogen transmission can occur through ingestion of contaminated drinking water or direct contact with flood waters. Due to these mechanisms, diarrheal and gastrointestinal (GI) illnesses are among the more common diseases noted after floods. The relatively short lag period between flooding and increases in GI illness noted in multiple studies indicated a viral infection due to direct contact with contaminated flood water (Ding et al. 2013; Wade et al. 2004, 2014). Other viral GI pathogens such as norovirus have been linked to outbreaks due to direct contact with sewage contaminated flood waters (Schmid et al. 2005). Illnesses such as hepatitis A, bacillary dysentery, and diarrhea were also hypothesized to be due to direct exposure to floodwaters or contaminated drinking water (Gao et al. 2016). A study of typhoid in Dhaka, Bangladesh showed that cases increase geographically around rivers and temporally after heightened rainfall and river levels (Dewan et al. 2013). Disease risk can also be modified by water source and possible disruption and changes in water source as a result of flooding. Kazama et al. (2012) showed risk of GI illness was inversely related to flood size in residential areas with smaller floods conferring greater risk than larger floods. The risk of infection was also mediated by water source, with greater risk from groundwater sources than surface water sources in sparsely populated regions (Kazama et al. 2012).

The effect of flooding on diarrheal illness is subject not only to the severity of the flood but the weather status prior to the flood. Heavy rainfall following dry periods could pose greater risk of diarrheal illness than continuous periods of wet weather (Carlton et al. 2014). A study of recurrent floods in India showed that long-term impacts of seasonal flooding are not as significant as that of sporadic flooding on childhood diarrheal illnesses (Joshi et al. 2011). It is possible that in contrast to sporadic flooding, seasonal floods are predictable dangers in some regions and preparations can be made to avoid related illnesses. Extreme flooding has been reported as a risk factor for cholera outbreaks in many regions as well (Griffith et al. 2006). Dual peaks in cholera occurrence in the Bengal delta were explained by both droughts and floods in the region (Akanda et al. 2009). Two studies following illness after consecutive major floods in Bangladesh showed variation in the causative pathogens of diarrhea by flood with the most common pathogen being *Vibrio cholerae* followed by rotavirus. Differences among the floods could be due to the natural seasonality of the diseases and other secular trends in healthcare occurring at the time of flood (Harris et al. 1998; Schwartz et al. 2006).

Incidences of disease which occur after flooding may be contracted through routes of exposure besides drinking water such as direct contact with floodwaters, where pathogens can enter the body through exposed or broken skin. A study of the health effects associated with the 2013 Alberta (Canada) floods revealed increases in tetanus shots and injuries associated with flooding (Sahni et al. 2016). Depending on the setting and the ability of the population to avoid the inundated area during the flood, it is possible that the majority of this direct contact risk comes from the clean-up process and not the initial inundation phase of the flood (Fewtrell et al. 2011). Direct exposure to flood waters can also lead to outbreaks in certain zoonotic disease such as leptospirosis in endemic Southeast Asian and south/central American countries, with municipalities lying in floodplains often correlated with higher rates of disease (Barcellos and Sabroza 2001; de Resende et al. 2016).

Floods can also indirectly impact human health by supporting or spreading breeding grounds and dispersal of pathogen vectors. Flooding along the Yangtze River, China corresponded with the spread of schistosomiasis carrying snails to previously disease-free areas. Cases of schistosomiasis among humans and animals rose after a large flood in the area and the highest rates were localized to lakeside provinces along the Yangtze (Wu et al. 2008). Malaria was found to increase after extreme flooding in multiple studies due to the creation of stagnant pools of water that are necessary breeding grounds for the mosquitoes that carry and spread the pathogen. Boyce et al. (2016) showed malaria rates increased by 30% in areas bordering a recently flooded river. This spike in morbidity occurred at a time that was uncharacteristic for malaria season and was attributed to the flood waters creating stagnant waters for breeding that otherwise would not be present (Boyce et al. 2016). A temporal analysis of malaria after extreme flooding showed peak malaria rates at 25 days post-flood, consistent with the delay expected for mosquito growth, disease transmission and presentation (Ding et al. 2014). This lag period is much longer than that associated with viral GI illness and raises the issue of identifying an appropriate surveillance period when monitoring flood-related disease outbreaks. For certain diseases, a flood-related event might not show increases in cases until weeks after the flood has receded, especially if the organisms are able to remain in the soil. An outbreak of cryptosporidium among children in Halle, Germany was linked to their participation in activities on a floodplain 2 weeks after flood waters had receded and the floodplain had been reopened to the public (Gertler et al. 2015).

It is clear that flooding has important impacts on infectious disease but future research is needed on the relationship between flood size, flood occurrence, environmental conditions, and risk of health impacts. Unfortunately, many other methodological issues continue to complicate our understanding of the links between flood events and disease. Improved disease surveillance and flooding impact assessments need to be made, with better record keeping and sharing between government, relief, and other agencies involved in flood response. The disruptive nature of flood events can limit access to hospitals, possibly resulting in underestimates of disease rates if using hospital admission data or other forms of passive surveillance. Certain disease outcomes such as GI illness often may not require an ER visit or hospitalization which could also lead to underestimates of disease rates after flooding. Studies are also often correlative. Correlation analyses could be exposing direct relationships between flooding and disease or possible indirect relationships due to associations between flood risk areas and susceptible or high-risk populations. Extreme weather events convey a risk with respect to waterborne diseases and will disproportionately impact sectors of populations with preexisting health problems (Cann et al. 2013) and which lack preparedness (Sahni et al. 2016). Very large floods can also act to concentrate the population in areas with polluted water and poor hygiene services (Griffith et al. 2006). Although impacts are not limited to regions with poor services (e.g., treatment (Charron et al. 2004; Wade et al. 2014)), the impact of floods on waterborne outbreaks will be modulated by the population density, underlying health status, and availability of health care (Watson et al. 2016). A better understanding of how floods can negatively affect health can also aid in prevention methods such as prophylaxis or vaccination campaigns against certain diseases that might increase in incidence after flooding (Dechet et al. 2012; Wu et al. 2008). Finally, future studies should pay special attention to any differential health effects that can arise from sporadic flooding compared to seasonal rains (e.g. monsoons) and associated flooding.

### Climate regulation

Floods impact heterotrophic processes tied to the production and consumption of greenhouse gases (GHG: CO_2_, CH_4_, and to some extent N_2_O) as a climate regulating ecosystem service provided naturally by soil systems. These processes include aerobic respiration of a wide range of organic compounds in floodwater (produces CO_2_), methanogenesis (produces CH_4_), and methane-oxidation (consumes CH_4_). Other processes (e.g. acetate reduction) can produce CO_2_ but are secondary in soil and will therefore not be discussed here. The primary process tied to N_2_O production in soils is heterotrophic denitrification, or the reduction of NO_3_^−^ into N_2_ gas, which when incomplete leads to the production of N_2_O gas (Naiman et al. 2005). Increased nitrogen supply during flooding may provide the raw materials for denitrification, but N_2_O production is generally small in floodplains (Kaushal et al. 2014). Additionally, N_2_O production following flooding is variable and relies on inundation time, substrate, and temperature (Kaushal et al. 2014; Pinay et al. 2002). A thorough review of the conditions (e.g., temperature, moisture availability, electron donors and acceptors) regulating these processes and associated GHG consumption or production can be found in Schlesinger and Bernhardt (2013). In addition to soil processes, flooding can transport large amounts of soil organic matter into aquatic ecosystems, where it can be processed further and release CO_2_ (Richey et al. 2002).

Although translating changes in GHG fluxes at the soil-atmosphere interface into a single variable of air quality regulation remains a challenge, many studies have documented how GHG fluxes change in response to floods and water pulses at the soil-atmosphere interface (Kim et al. 2012). Although many more studies should be conducted to fully comprehend how GHG fluxes and associated air quality ecosystem services change following flooding events, some trends can be identified from published studies. In water limited environments where aerobic respiration is often limited by water availability, water additions/small floods generally lead to increased CO_2_ emissions (Leon et al. 2014), but no consistent response across systems with respect to N_2_O and CH_4_. In a xeric environment (AZ, USA), Harms and Grimm (2012) show that following dry antecedent conditions, small floods typically stimulated CO_2_ and CH_4_ production, but not N_2_O production. In wet and non-water limited environments, flood events typically lead to enhanced N_2_O and CH_4_ fluxes, especially under warm temperature conditions (> 20 °C). Under wet antecedent conditions (monsoon season), muted CO_2_ and N_2_O responses were observed, while CH_4_ emission increased following water additions (Harms and Grimm 2012). On the other hand, CO_2_ fluxes under these conditions generally do not change drastically following storms as they mostly vary on a seasonal basis with higher CO_2_ fluxes during summer months. In central New York state, USA, the remnants of Hurricane Irene and Tropical Storm Lee caused a large flood, which increased N_2_O flux from 0.2 to 1.49 mg N/m^2^/day and CH_4_ flux from a range between − 2 and 2 mg C/m^2^/day pre-flood to 2.76 mg C/m^2^/day post-flood, and increased short pulses in CO_2_ at the onset of precipitation (Vidon et al. 2016a). In a water-limited forested riparian zone in North Carolina, USA, Vidon et al. (2016b) reported less negative CH_4_ fluxes (i.e., methane oxidation decreased) and higher CO_2_ fluxes (i.e., aerobic respiration increased) following water additions.

From an ecosystem services perspective, this suggests that if flood events become more frequent, ecosystems may present higher overall efflux of GHGs (Petrakis et al. 2018). Indeed, as indicated above, in water-limited environments, higher CO_2_ production and associated emissions are likely to lead to overall increases in GHG emissions. In wetlands where strong CH_4_ responses to storms are observed and where CH_4_ can contribute large fractions of total GHG, an increased frequency in floods will also likely lead to overall increases in total GHG fluxes (e.g., Gomez et al. 2016). Finally, in hay and fertilized cornfields where CH_4_ and N_2_O combined can represent approximately 50% of total CO_2_ emissions, floods are also likely to lead to overall increased GHG emissions (Bressler et al. 2017). It is only in non-water limited environments where most CO_2eq_ fluxes are generated by CO_2_ emissions that floods are unlikely to have any significant impact on total GHG fluxes, as only muted CO_2_ responses to storms are observed in these environments. Overall, climate and land use are therefore key factors to consider in assessing how floods might impact ecosystem services related to GHG induced changes in climate.

## Provisioning services

### Drinking water

Floods can impact drinking water when contaminants and pathogens are discharged into surface and underground drinking water sources. Any pollutants that are mobilized during flooding can impact drinking water sources. For example, flooding can increase total coliform (TC) concentrations by suspending sediment containing coliforms in rivers (Smith et al. 2008) or causing waste water from flooded sewer systems to infiltrate drinking supplies (Islam et al. 2007). Human wastes can also quickly infiltrate drinking water supplies during flooding in areas that lack proper waste disposal (Zahoor et al. 2016). Additionally, animal wastes can contaminate drinking water by contributing nutrients, pathogens, and metals (Burkholder et al. 2007). Metals stored in sediment can also be resuspended in aquatic ecosystems or enter drinking water sources through connectivity with contaminated water or runoff (Chrastny et al. 2006). Therefore, flooding has the potential to negatively impact drinking water supplies in a variety of ways.

For our literature survey, we considered a mixture of drinking water sources including drinking water reservoirs, wells, and taps. Here, we used TC and metal concentrations to assess the effects of flooding on drinking water. Limits on these parameters are among many criteria set for drinking water but are the most commonly reported in the literature. Nevertheless, TC and metal concentrations were only reported in the literature for extreme flooding. Therefore, we also included studies which quantified herbicides in drinking water supplies following flooding, including one study which quantified the herbicide atrazine after a small flood. These parameters were also included because they have significant health impacts when concentrations exceed drinking water standards. Bacteria present in drinking water can cause illnesses and even death in high-risk age groups such as children and the elderly (Figueras and Borrego 2010). Metal ingestion can have effects on the immune system, blood, liver, kidneys, and nervous system (Cempel and Nikel 2006).

In most studies, the quality of drinking water sourced from the tap or well water decreased after extreme flooding events. TC counts were compared to either local or more commonly World Health Organization (WHO) standards. Almost all well and tap water sampled after extreme flooding contained TC concentrations that exceeded drinking water standards (e.g. Chaturongkasumrit et al. 2013; Eccles et al. 2017; Islam et al. 2007). Metal concentrations measured included chromium, nickel, iron, lead, and cadmium. Most post-flood metal concentrations were elevated beyond pre-flood values in well and tap water (Zahoor et al. 2016) and in a drinking water reservoir (Chrastny et al. 2006). However, lead concentrations remained below World Health Organization (WHO) water quality standards after flooding in Lower Pakistan (Zahoor et al. 2016).

There were no results for the impact of small floods on either TC or metal concentrations. However, one study measured concentrations of the herbicide atrazine in drinking water sources following small floods. Small floods did not increase atrazine levels in drinking water supplies (Ray et al. 2002). Concentrations of the herbicides atrazine, alachlor, and cyanazine in well water also did not increase after extreme flooding (Chong et al. 1998). However, these results are influenced by the timing of herbicide application relative to the flood events. Flooding will likely mobilize recently applied herbicides from agricultural land and contaminate drinking water sources. One additional study which, used a water quality index found that drinking water quality decreased following seasonal flooding (Chen et al. 2015). Small floods can negatively impact drinking water, but there is a lack of evidence in this area to indicate the scope or prevalence of such impacts.

### Food supply

Food sources that may be affected by flooding include fish, livestock, and crops. Flooding can increase soil regeneration and water availability for agriculture (Ogbodo 2011) or livestock and increase fish habitat and availability of food sources for fish (Jellyman et al. 2013). Small or seasonal flooding also is advantageous for native fish populations relative to invasive fishes occupying the same areas (Ho et al. 2013). However, extreme floods can destroy planted crops (Ferguson et al. 2012), drown livestock (Atta-ur-Rahman 2013), and impair fish catch by reducing fish density (Endo et al. 2016). Fish production may increase or stay constant if an extreme flood falls within the normal flood regime that individual fishes are adapted to (Lytle and Poff 2004; Poff et al. 1997). Flood impact on fish populations is further complicated by flood timing. Floods that inundate large areas and occur when temperatures are warm are likely to result in hypoxia, affecting fish physiology, behavior, and survival (Pasco et al. 2016). Additionally, small floods that occur when temperatures are too low for native fish spawning may cause proliferation of invasive fish populations (Rayner et al. 2015). Communities which rely on subsistence farming and fishing are especially vulnerable to food reduction during and after flooding.

Most surveyed studies reported negative effects of extreme flooding on food supply. Several studies reported that crops were damaged during extreme flooding and that such flooding caused significant hardships for people who relied on farming as their main food source. Additionally, if extreme flooding extended into the next planting season farmers lost additional crops (Haile et al. 2013). Extreme flooding increased fish availability when floodwaters rose and receded. However, fewer fish were available when floodwaters were high (Sherman et al. 2015). In all studies, fish catch and consumption patterns were similar during small floods. People generally caught and consumed the least amount of fish during high water compared to periods of rising and receding floodwater (Isaac et al. 2015; Endo et al. 2016). Very few studies reported on flood impacts on livestock; however, one study reported that over 52,000 cattle drowned following an extreme flooding event that occurred in 2010 in Khyber Pakhtunkhwa, Pakistan (Atta-ur-Rahman 2013). Therefore, extreme flooding negatively impacts food sources such as crops, fish, and livestock. There was an inadequate number of studies to determine the effects of small floods on agriculture. However, small floods should have either a net neutral or positive effect on agriculture due to increased water availability, more nutrients, and enhanced soil renewal processes (Ogbodo 2011). The importance of fish and crops as food sources differs depending on the society’s location making it difficult to compare the relative importance of flooding. The effects of flooding on food supply also differ depending on the food source considered and at which stage of flooding food sources are quantified. For example, fish catch decreased during high water, but increased as water receded in a village on the banks of the Peruvian Amazon (Sherman et al. 2015). However, high water lasted months in some cases which was detrimental to people who rely on fish as a major part of their diets.

## Cultural services

### Aesthetic value

Aesthetic value refers to the view and natural qualities near water bodies that people find desirable. A flood, whether minor or major, can physically and functionally modify the ecosystem and infrastructure, which usually results in a reduction of the aesthetic value. Over longer term between extreme flood events, the aesthetic value generally recovers or can even be increased above the pre-flood value, depending upon the nature of the post-flood ecosystem recovery or shifts (e.g. Ronnback et al. 2007) and the implementation of post-flood management practices. Flood zone property values are generally enhanced by higher aesthetic value, but property values are also reduced by the perceived risk of floods (e.g. Shilling et al. 1985; MacDonald et al. 1987).

There was a lack of evidence for small floods affecting housing value, but extreme flooding led to decreased housing values in all cases. Home prices decreased markedly immediately following a flood event, particularly for lower priced properties in the 100-year flood plain, or in neighborhoods directly damaged by the flood (e.g. Bin and Polansky 2004; Eves and Wilkinson 2014). In contrast, higher priced properties in the 500-year flood plain were not found to decrease in value following a flood (Shultz and Fridgen 2001). This is attributed to a lack of awareness of home owners to the risks associated with the 500-year flood plain.

### Recreation and tourism

Recreation refers to leisure activities that typically include fishing, boating, swimming, hunting, and hiking. Increases in river discharge can impact these activities by reducing safety with high flows and impaired water quality. However, higher water levels can also lead to enhanced fishing (Miranda and Meals 2013) and boating conditions (Stewart et al. 2003). The magnitude of flooding determines the effects on recreation. Major floods have a very immediate negative effect on recreation activities due to physical damage to infrastructure, ecosystems, and the loss of aesthetic value (Burger 2015). The long-term impact of a major flood on recreation is varied and depends strongly on the post-flood control and management of both information and recovery efforts. Tourism or ecotourism is related to recreation, but involves people traveling from outside the region, which generates additional economic value to nearby communities. Flooding may impact tourism by damaging infrastructure, reducing safety, damaging sites of interest, and changing tourist perceptions of an area (Walters et al. 2015).

From our literature review, we found that recreation is negatively impacted by extreme flooding. People were less likely to visit a recreational site, such as a park, after extreme flooding had occurred (Rung et al. 2011). Small floods had a general positive impact on recreation. Small experimental floods increased recreation by increasing the size and number of sandbars suitable for boats to stop at below the Glen Canyon Dam, Arizona (Stewart et al. 2003). Additionally, one study found that a study group comprised of students preferred rivers and streams located within parks to have dynamic hydrology (Eder and Arnberger 2016). Therefore, people are more likely to recreate in parks where natural water features have dynamic hydrology. Small floods increase hydrologic variability without causing the damages associated with extreme flooding. The effects of extreme flooding on tourism were mixed. Negative impacts included revenue losses (Kala 2014), evacuations (Faulkner and Vikulav 2001), and tourists deciding to avoid visiting the flooded area (Walters et al. 2015). These effects were temporary and tourism returned to pre-flood values after flood waters receded. In one study, tourists simply rescheduled their trips instead of traveling to an unaffected area (Faulkner and Vikulav 2001). It was also reported that flooded areas can appeal to travelers who want to help those affected (Walters et al. 2015). We were unable to make any conclusions on the impacts of small floods on tourism since we found no literature. However, there is some evidence that people tend to desire visiting areas with dynamic river systems so small floods may enhance tourism. As with recreation, the post-flood recovery efforts and the message communicated to the public play a crucial role (e.g. Walters et al. 2015). Education of the public through media presentations and outreach activities is very influential in restoring recreational activities. Having a disaster preparedness plan prior to an extreme flood, with effective implementation following a flood, can significantly improve the post-flood recovery in recreational and tourist activity (Faulkner and Vikulov 2001).

## Conclusions

The influence of flooding on ecosystem services depends on flood size and service type with extreme floods more likely to be associated with declines in ecosystem services whereas small floods provide or enhance many ecosystem services (Fig. 1; Table 3). Although we detected trends in ecosystem service availability following flooding, many services responded in complicated ways. Initial aquatic ecosystem conditions and time of year were important for determining whether a flood event, extreme or small, would result in gains or losses of a given ecosystem service. For example, floods occurring during warmer months with good light conditions were capable of causing algal blooms. However, a flood of the same size occurring in a different season may have no effect on primary production due to light limitation. Future research on the nuances involved with producing the ecosystem services addressed in this study should be done to improve our understanding of these services and how disturbances will affect them. Additionally, studies linking ecosystem processes with ecosystem services should be undertaken to improve our understanding of the effects of disturbance on aquatic ecosystem services in general.

River flooding is an essential component of natural flow regimes. However, against the backdrop of human-dominated systems, extreme floods were almost exclusively negatively associated with post-flood changes in aquatic ecosystem services (Table 3). More frequent extreme flooding will likely exacerbate losses in ecosystem services and possibly leave inadequate time for recovery between flood events. Ecosystem recovery following extreme floods is highly variable and can last months to years, depending on the effect considered (Swanson et al. 1998). For example, contaminant pulses resulting from extreme floods can be elevated for days to years post-flood (Kaushal et al. 2014). It is difficult to estimate ecosystem service recovery time following floods because monitoring typically does not extend beyond one post-flood measurement. Additionally, larger changes from pre- to post-flood could extend recovery time. Losses in ecosystem services such as drinking water and food supply will be especially detrimental in areas that lack drinking water filtration facilities (Delpla et al. 2009) and rely on subsistence farming (Haile et al. 2013) and fishing for food (Sherman et al. 2015). Approaches to reduce flood impacts on ecosystem services could include relocating agricultural land further from flood prone areas when possible, reducing impervious surfaces near water, reducing point and nonpoint pollution sources, and restoring riparian zones (Kaushal et al. 2014). However, there is much more work that needs to be done to find effective ways to manage extreme flooding.

Small floods were more likely to be associated with positive or neutral effects on ecosystem services (Table 3). However, small floods negatively affected water quality and disease regulation, but post-flood recovery may occur quickly because the magnitude of ecosystem service change following small floods is generally small compared to extreme floods. Additionally, these smaller floods typically occur seasonally and aquatic ecosystems are usually well-adapted to these disturbances (Junk et al. 1989). Many aquatic ecosystems do not experience these small beneficial floods because of damming and water regulating structures (Death et al. 2015), so there is no opportunity for flooding to enhance ecosystem service provisioning. Therefore, small floods should be favored as part of a healthy flow regime in aquatic ecosystems. Preserving natural flow variation that contributes to small floods is important for aquatic ecosystems and as shown here, ecosystem service provision. Activities which preserve the occurrence of small floods include decreasing impervious surfaces and restoring riparian areas to reduce runoff that increases flood magnitude (Ogden et al. 2011) and limiting the extent of flow alteration such as refraining from building dams (Acreman et al. 2014).

Many previous studies have reported that dynamic flow regimes that include floods, even occasional extreme floods, are ecologically important (Peters et al. 2016) but few have linked floods with aquatic ecosystem service provisioning. We evaluated ecosystem service gains and losses in response to flooding and identified possible mechanisms that lead to these changes (Fig. 3) and found that aquatic ecosystems require flood protection strategies designed to dampen the undesired effects of extreme floods and enhance smaller beneficial floods to maximize ecosystem service provision. There are many methods available to do this including restoring lateral connectivity between the river and floodplain, regenerating functional riparian areas (Death et al. 2015), reconnecting fragmented aquatic ecosystems to reduce runoff, and reforesting headwaters (Barbedo et al. 2014). Not all floods can or should be prevented, but these strategies in combination should improve flood regulation without exerting the negative impacts commonly associated with flood mitigation practices. However, we must be diligent in designing and implementing these plans as quickly as possible because current and future increases in flood magnitude will be deleterious to aquatic ecosystems and reduce aquatic ecosystem services. Ecosystem services examined in this study represent some of the essential life sustaining benefits that people gain from aquatic ecosystems such as food supply, drinking water, and human disease regulation. Flood protection strategies that are effective at reducing the damages caused by extreme flooding will have profound benefits beyond protecting our built infrastructure. They will also protect the aquatic ecosystems and their ecosystem services that we rely on for health and survival.

## Electronic supplementary material

Below is the link to the electronic supplementary material.

Supplementary material 1 (DOCX 24 kb)
