# Supplementary material for: The impact of flooding on aquatic ecosystem services
Source: Biogeochemistry. 2018 May 11;141(3):439–61. doi: 10.1007/s10533-018-0449-7 (PMC6404734; doi:10.1007/s10533-018-0449-7)
Supplement: Supplementary file 1 — Supplementary material 1 (DOCX 24 kb) [file 10533_2018_449_MOESM1_ESM.docx]

**Appendix 1**

Table 1. Number of sources found and country where each study was completed for each ecosystem service and flood size.

| Service | Flood size | Number of papers | Country (no. papers) |
| --- | --- | --- | --- |
| Primary production | small | 8 | Australia (1), Botswana (1), Brazil (1), Peru (1), Spain (1), Switzerland (2), USA (2), Venezuela (1) |
|  | extreme | 3 | Botswana (1), Brazil (1), USA (1) |
| Soil formation | small | 6 | Australia (1), Bangladesh (1), Cambodia (1), India (1), Pakistan (1), USA (1) |
|  | extreme | 8 | Bangladesh (1), Canada (1), France (2), New Zealand (1), Pakistan (2), USA (1) |
| Water regulation | small | 11 | Australia (1), Cambodia (1), China (1), Ethiopia (1), Mali (1), Namibia (3), Niger (1), Spain (1), Turkey (1), USA (1) |
|  | extreme | 6 | Australia (1), Chile (1), China (1), Namibia (1), Spain (1), USA (1) |
| Water quality | small | 8 | Brazil (1), China (1), Croatia (1), France (1), Italy (1), Morocco (1), USA (2) |
|  | extreme | 8 | Austria (1), Germany (1), Poland (1), Taiwan (1), USA (4) |
| Regulation of human disease | small | 3 | Cambodia (1), China (1), Sudan (1) |
|  | extreme | 12 | Bangladesh (2), Cambodia (1), Canada (1), China (3), Germany (1), Sudan (1), Uganda (1), USA (2) |
| Climate regulation | small | 11 | Austria (1), Botswana (1), Brazil (4), Spain (1), USA (3), Venezuela (1) |
|  | extreme | 2 | Botswana (1), USA (1) |
| Drinking water | small | 2 | China (1), USA (1) |
|  | extreme | 8 | Australia (1), Bangladesh (1), Canada (1), Czech Republic (1), Germany (1), Pakistan (1), Thailand (1), USA (1) |
| Food supply | small | 3 | Brazil (2), Pakistan (1) |
|  | extreme | 6 | Bangladesh (1), Ethiopia (1), India (1), Mozambique (1), Pakistan (1), Peru (1) |
| Aesthetic value | small | none | None |
|  | extreme | 5 | Australia (1), USA (4) |
| Recreation and tourism | small | 2 | Germany (1), USA (1) |
|  | extreme | 4 | Australia (2), India (1), USA (1) |

**References Appendix 1**

Abril G, Martinez J-M, Artigas LF, et al (2013) Amazon River carbon dioxide outgassing fuelled by wetlands. Nature 505:395–398

Acuna V, Giorgi A, Munoz I, et al (2007) Meteorological and riparian influences on organic matter dynamics in a forested Mediterranean stream. J North Am Benthol Soc 26:54–69

Akanda AS, Jutla AS, Islam S (2009) Dual peak cholera transmission in Bengal Delta: A hydroclimatological explanation. Geophys Res Lett 36:1–6

Aksoy H, Wittenberg H (2015) Baseflow Recession Analysis for Flood-Prone Black Sea Watersheds in Turkey. Clean - Soil, Air, Water 43:857–866

Amiaz Y, Sorek S, Enzel Y, Dahan O (2011) Solute transport in the vadose zone and groundwater during flash floods. Water Resour Res 47:1–10

Aryal R, Grinham A, Beecham S (2014) Tracking Inflows in Lake Wivenhoe during a Major Flood Using Optical Spectroscopy. Water 6:2339–2352

Ashraf M, Bhatti MT, Shakir AS (2016) River bank erosion and channel evolution in sand-bed braided reach of River Chenab: role of floods during different flow regimes. Arab J Geosci 9:140

Atta-ur-Rahman, Khan AN (2011) Analysis of flood causes and associated socio- economic damages in the Hindukush region. Nat Hazards 59:1239–1260

Atta-ur-Rahman, Khan AN (2013) Analysis of 2010-flood causes, nature and magnitude in the Khyber Pakhtunkhwa, Pakistan. Nat Hazards 66:887–904

Bales JD (2003) Effects of Hurricane Floyd inland flooding, September–October 1999, on tributaries to Pamlico Sound, North Carolina. Estuaries and Coasts 26:1319– 1328

Bartlett KB, Crill PM, Bonassi JA, et al (1990) Methane flux from the Amazon River floodplain: Emissions during rising water. J Geophys Res 95:16773–16778

Belger L, Forsberg BR, Melack JM (2011) Carbon dioxide and methane emissions from interfluvial wetlands in the upper Negro River basin, Brazil. Biogeochemistry 105:171–183

Bianchi TS, Freer ME, Wetzel RG (1996) Temporal and spatial variability, and the role of dissolved organic carbon (DOC) in methane fluxes from the Sabine River floodplain (southeast Texas, USA). Arch Fur Hydrobiol 136:261–287

Boyce R, Reyes R, Matte M, et al (2016) Severe Flooding and Malaria Transmission in the Western Ugandan Highlands: Implications for Disease Control in an Era of Global Climate Change. J Infect Dis 214:1403–1410

Carroll RWH, Warwick JJ, James AI, Miller JR (2004) Modeling erosion and overbank deposition during extreme flood conditions on the Carson River, Nevada. J Hydrol 297:1–21

Chadwick MT, Soussan JG, Martin TC, et al (2001) Bank robbery: The real losers in the 1998 Bangladesh flood. L Degrad Dev 12:251–260

Chaturongkasumrit Y, Techaruvichit P, Takahashi H, et al (2013) Microbiological evaluation of water during the 2011 flood crisis in Thailand. Sci Total Environ 463–464:959–967

Chen YC, Liu JH, Kuo JT, Lin CF (2013) Estimation of phosphorus flux in rivers during flooding. Environ Monit Assess 185:5653–5672

Chen Y, Song X, Zhang Z, et al (2015) Simulating the impact of flooding events on non- point source pollution and the effects of filter strips in an intensive agricultural watershed in China. Limnology 16:91–101

Chen Z, Zhu Z, Yin L, et al (2015) The Changing Water Quality Characteristics from Urban Drinking Water Sources in Guangdong, China. Water Resour Manag 29:987–1002

Cherifi O, Loudiki M (1999) Flood transport of dissolved and suspended matter in the El Abid river basin (Morocco). Hydrobiologia 410:287–294

Chong SK, Klubek BP, Weber JT (1998) Herbicide contamination by the 1993 great flood along the Mississippi River. J Am Water Resour Assoc 34:687–693

Crase L, Gillespie R (2008) The impact of water quality and water level on the recreation values of Lake Hume. Australas J Environ Manag 15:21–29

Dahan O, Tatarsky B, Enzel Y, et al (2008) Dynamics of Flood Water Infiltration and Ground Water Recharge in Hyperarid Desert. Ground Water 46:450–461

Daniel VE, Florax RJGM, Rietveld P (2009) Flooding risk and housing values: An economic assessment of environmental hazard. Ecol Econ 69:355–365

Dewan A, Corner R, Saleem A, et al (2017) Geomorphology Assessing channel changes of the Ganges-Padma River system in Bangladesh using Landsat and hydrological data. Geomorphology 276:257–279

Ding G, Gao L, Li X, et al (2014) A Mixed Method to Evaluate Burden of Malaria Due to Flooding and Waterlogging in Mengcheng County, China: A Case Study. PLoS One 9:1–10

Ding G, Zhang Y, Gao L, et al (2013) Quantitative Analysis of Burden of Infectious Diarrhea Associated with Floods in Northwest of Anhui Province, China: A Mixed Method Evaluation. PLoS One 8:1–9

Eccles KM, Checkley S, Sjogren D, et al (2017) Lessons learned from the 2013 Calgary flood: Assessing risk of drinking water well contamination. Appl Geogr 80:78–85

Eder R, Arnberger A (2016) How heterogeneous are adolescents’ preferences for natural and semi-natural riverscapes as recreational settings? Landsc Res 41:555–568

Endo W, Peres CA, Haugaasen T (2016) Flood pulse dynamics affects exploitation of both aquatic and terrestrial prey by Amazonian floodplain settlements. Biol Conserv 201:129–136

Eves C, Wilkinson S (2014) Assessing the immediate and short-term impact of flooding on residential property participant behaviour. Nat Hazards 71:1519–1536

Faulkner B, Vikulov S (2001) Katherine, washed out one day, back on track the next: a post-mortem of a tourism disaster. Tour Manag 22:331–344

Ferguson ME, Jones RB, Bramel PJ, et al (2012) Post-flooding disaster crop diversity recovery: a case study of Cowpea in Mozambique. Disasters 36:83–100

Fridgen PM, Shultz SD (1999) The Influence of the Threat of Flooding on Housing Values in Fargo, North Dakota and Moorhead, Minnesota. Agric Econ Rep

Fuller IC (2008) Geomorphic impacts of a 100-year flood: Kiwitea Stream, Manawatu catchment, New Zealand. Geomorphology 98:84–95

Gammons CH, Shope CL, Duaime TE (2005) A 24 h investigation of the hydrogeochemistry of baseflow and stormwater in an urban area impacted by mining: Butte, Montana. Hydrol Process 19:2737–2753

Gao L, Zhang Y, Ding G, Liu Q (2016) Projections of hepatitis A virus infection associated with flood events by 2020 and 2030 in Anhui Province, China. Int J Biometeorol 60:1873–1884

Gertler M, Dürr M, Renner P, et al (2015) Outbreak of cryptosporidium hominis following river flooding in the city of Halle (Saale), Germany, August 2013. BMC Infect Dis 15:1–10

Gotkowitz MB, Attig JW, McDermott T (2014) Groundwater flood of a river terrace in southwest Wisconsin, USA. Hydrogeol J 22:1421–1432

Grove JR, Croke J, Thompson C (2013) Quantifying different riverbank erosion processes during an extreme flood event. Earth Surf Process Landforms 38:1393–1406

Haile AT, Kusters K, Wagesho N (2013) Loss and damage from flooding in the Gambela Region, Ethiopia. Int J Glob Warm 5:483–497

Hassan OA, Ahlm C, Sang R, Evander M (2011) The 2007 Rift Valley Fever Outbreak in Sudan. PLoS Neglected Trop Dis 5:1–7

Houston J (2006) The great Atacama flood of 2001 and its implications for Andean hydrology. Hydrol Process 20:591–610

Hubbard L, Kolpin DW, Kalkhoff SJ, Robertson DM (2011) Nutrient and Sediment Concentrations and Corresponding Loads during the Historic June 2008 Flooding in Eastern Iowa. J Environ Qual 40:166–175

Isaac VJ, Almeida MC, Giarrizzo T, et al (2015) Food consumption as an indicator of the conservation of natural resources in riverine communities of the Brazilian Amazon. An Acad Bras Cienc 87:2229–2242

Islam MS, Brooks A, Kabir MS, et al (2007) Faecal contamination of drinking water sources of Dhaka city during the 2004 flood in Bangladesh and use of disinfectants for water treatment. J Appl Microbiol 103:80–87

Jacinthe PA (2015) Carbon dioxide and methane fluxes in variably-flooded riparian forests. Geoderma 241:41–50

Jacob J, Sanders T, Dähnke K (2016) Nitrite consumption and associated isotope changes during a river flood event. Biogeosciences 13:5649–5659

Kala CP (2014) Deluge, disaster and development in Uttarakhand Himalayan region of India: Challenges and lessons for disaster management. Int J Disaster Risk Reduct 8:143–152

Kazama S, Aizawa T, Watanabe T, et al (2012) A quantitative risk assessment of waterborne infectious disease in the inundation area of a tropical monsoon region. Sustain Sci 7:45–54

Kazama S, Hagiwara T, Ranjan P, Sawamoto M (2007) Evaluation of groundwater resources in wide inundation areas of the Mekong River basin. J Hydrol 340:233–243

Keilholz P, Disse M, Halik Ü (2015) Effects of land use and climate change on groundwater and ecosystems at the middle reaches of the Tarim River using the MIKE SHE integrated hydrological model. Water 7:3040–3056

Kistemann T, Claßen T, Koch C, et al (2002) Microbial Load of Drinking Water Reservoir Tributaries during Extreme Rainfall and Runoff. Appl Environ Microbiol 68:2188–2197

Kousky C (2010) Learning from Extreme Events: Risk Perceptions after the Flood. Land Econ 86:395–422

Kunii O, Nakamura S, Abdur R, Wakai S (2002) The impact on health and risk factors of the diarrhoea epidemics in the 1998 Bangladesh floods. Public Health 116:68–74

Laignel B, Dupuis E, Durand A, et al (2006) Erosion balance in the watersheds of the western Paris Basin by high-frequency monitoring of discharge and suspended sediment in surface water. Comptes Rendus Geosci 338:556–564

Leyland J, Hackney CR, Darby SE, et al (2017) Extreme flood-driven fluvial bank erosion and sediment loads: direct process measurements using integrated Mobile Laser Scanning (MLS) and hydro-acoustic techniques. Earth Surf Process Landforms 42:334–346

Lindholm M, Hessen DO, Mosepele K, Wolski P (2007) Food webs and energy fluxes on a seasonal floodplain: The influence of flood size. Wetlands 27:775–784

Lutz A, Thomas JM, Panorska A (2011) Environmental controls on stable isotope precipitation values over Mali and Niger, West Africa. Environ Earth Sci 62:1749– 1759

Mccallum AM, Andersen MS, Acworth RI (2014) A New Method for Estimating Recharge to Unconfined Aquifers Using Differential River Gauging. Groundwater 52:291–297

Morin E, Grodek T, Dahan O, et al (2009) Flood routing and alluvial aquifer recharge along the ephemeral arid Kuiseb River, Namibia. J Hydrol 368:262–275

Niemirycz E (1999) The Pollution Load from the River Odra in Comparison to That in Other Polish Rivers in 1988-1997. CLEAN - Soil, Air, Water 27:286–291

Oeurng C, Sauvage S, Sánchez-Pérez JM (2010) Temporal variability of nitrate transport through hydrological response during flood events within a large agricultural catchment in south-west France. Sci Total Environ 409:140–149

Ollivier P, Hamelin B, Radakovitch O (2010) Seasonal variations of physical and chemical erosion: A three-year survey of the Rhone River (France). Geochim Cosmochim Acta 74:907–927

Palijan G (2015) Towards deconfounding hydrological and seasonal temperature variability in the determination of selected limnological variables of a temperate floodplain ecosystem. Ecohydrology 8:325–339

Peng J, Ren Z, Song Y, et al (2015) Impact of spring flooding on DOM characterization in a small watershed of the Hun River, China. Environ Earth Sci 73:5131–5140

Pulliam WM (1993) Carbon Dioxide and Methane Exports from a Southeastern Floodplain Swamp. Ecol Monogr 63:29–53

Ramsankaran R, Kothyari UC, Ghosh SK, et al (2013) Physically-based distributed soil erosion and sediment yield model (DREAM) for simulating individual storm events. Hydrol Sci J 58:872–891

Rantala H, Glover D, Garvey J, et al (2016) Fish assemblage and ecosystem metabolism responses to reconnection of the Bird’s Point-New Madrid floodway during the 2011 Mississippi River flood. River Res Appl 32:1018–1029

Ray C, Soong TW, Lian YQ, Roadcap GS (2002) Effect of flood-induced chemical load on filtrate quality at bank filtration sites. J Hydrol 266:235–258

Roach KA, Winemiller KO, Davis SE (2014) Autochthonous production in shallow littoral zones of five floodplain rivers: Effects of flow, turbidity and nutrients. Freshw Biol 59:1–16

Rodrigues LC, Train S, Roberto M do C, Pagioro TA (2002) Seasonal fluctuation of some limnological variables on a floodplain lake (Patos lagoon) of the Upper Paraná River, Mato Grosso do Sul State, Brazil. Brazilian Arch Biol Technol 45:499–513

Rung AL, Broyles ST, Mowen AJ, et al (2011) Escaping to and being active in neighbourhood parks: park use in a post-disaster setting. Disasters 35:383–403

Saeck EA, Hadwen WL, Rissik D, et al (2013) Flow events drive patterns of phytoplankton distribution along a river- estuary-bay continuum. Mar Freshw Res 64:655–670

Sahni V, Scott AN, Beliveau M, et al (2016) Public health surveillance response following the southern Alberta floods, 2013. Can J Public Heal 107:142–148

Sánchez-Andrés R, Sánchez-Carrillo S, Ortiz-Llorente MJ, et al (2010) Do changes in flood pulse duration disturb soil carbon dioxide emissions in semi-arid floodplains? Biogeochemistry 101:257–267

Scofield V, Melack JM, Barbosa PM, et al (2016) Carbon dioxide outgassing from Amazonian aquatic ecosystems in the Negro River basin. Biogeochemistry 129:77–91

Sherman M, Ford J, Llanos-Cuentas A, et al (2015) Vulnerability and adaptive capacity of community food systems in the Peruvian Amazon: a case study from Panaillo. Nat Hazards 77:2049–2079

Sieczko AK, Demeter K, Singer GA, et al (2016) Aquatic methane dynamics in a human-impacted river-floodplain of the Danube. Limnol Oceanogr 61:S175–S187

Silva TSF, Melack JM, Novo EMLM (2013) Responses of aquatic macrophyte cover and productivity to flooding variability on the Amazon floodplain. Glob Chang Biol 19:3379–3389

Singh O, Singh H (2015) The response of farmers to the flood hazard under rice – wheat ecosystem in Somb basin of Haryana, India: an empirical study. Nat Hazards 75:795–811

Smith LK, Lewis WM, Chanton JP, et al (2000) Methane emissions from the Orinoco River floodplain, Venezuela. Biogeochemistry 51:113–140

Stewart W, Larkin K, Orland B, Anderson D (2003) Boater preferences for beach characteristics downstream from Glen Canyon Dam, Arizona. J Environ Manage 69:201–211

Stromberg JC, Richter BD, Patten DT, Wolden LG (1993) Response of a Sonoran riparian forest to a 10-year return flood. Gt Basin Nat 53:118–130

Tamminga AD, Eaton BC, Hugenholtz CH (2015) UAS-based remote sensing of fluvial change following an extreme flood event. Earth Surf Process Landforms 40:1464–1476

Tesi T, Miserocchi S, Acri F, et al (2013) Flood-driven transport of sediment, particulate organic matter, and nutrients from the Po River watershed to the Mediterranean Sea. J Hydrol 498:144–152

Uehlinger U (2000) Resistance and resilience of ecosystem metabolism in a flood-prone river system. Freshw Biol 45:319–332

Uehlinger U, Kawecka B, Robinson CT (2003) Effects of experimental floods on periphyton and stream metabolism below a high dam in the Swiss Alps (River Spöl). Aquat Sci 65:199–209

Vázquez-Suñé E, Capino B, Abarca E, Carrera J (2007) Estimation of recharge from floods in disconnected stream-aquifer systems. Groundwater 45:579–589

Wade TJ, Sandhu SK, Levy D, et al (2004) Did a Severe Flood in the Midwest Cause an Increase in the Incidence of Gastrointestinal Symptoms? Am J Epidemiol 159:398–405

Walraevens K, Vandecasteele I, Martens K, et al (2009) Groundwater recharge and flow in a small mountain catchment in northern Ethiopia. Hydrol Sci J 54:739–753

Walters G, Mair J, Ritchie B (2015) Understanding the tourist’s response to natural disasters: The case of the 2011 Queensland floods. J Vacat Mark 21:101–113

Wang X, Zhang G, Xu YJ (2015) Impacts of the 2013 extreme flood in Northeast China on regional groundwater depth and quality. Water 7:4575–4592

Weigelhofer G, Preiner S, Funk A, et al (2015) The hydrochemical response of small and shallow floodplain water bodies to temporary surface water connections with the main river. Freshw Biol 60:781–793

Weilhoefer CL, Pan Y, Eppard S (2008) The effects of river floodwaters on floodplain wetland water quality and diatom assemblages. Wetlands 28:473–486

Windle J, Rolfe J (2013) The impacts of the 2011 extreme weather events on holiday choices of Brisbane residents. Australas J Environ Manag 20:338–350

Wu J, Rees P, Dorner S (2011) Variability of E. coli density and sources in an urban watershed. J Water Health 9:94–106

Zahoor M, Khan FA, Azam M (2016) Bacteriological, inorganic and heavy metal evaluation of drinking water of the specified flood affected areas of Dir (Lower) Pakistan. Desalin Water Treat 57:13938–13957

Zhang L (2016) Flood hazards impact on neighborhood house prices: A spatial quantile regression analysis. Reg Sci Urban Econ 60:12–19

Zoboli O, Viglione A, Rechberger H, Zessner M (2015) Impact of reduced anthropogenic emissions and century flood on the phosphorus stock, concentrations and stocks in the Upper Danube. Sci Total Environ 518–519:117–129
